# 5-Cyclo­pentyl-2-methyl-3-(3-methyl­phenyl­sulfon­yl)-1-benzo­furan

**DOI:** 10.1107/S1600536814006187

**Published:** 2014-03-26

**Authors:** Hong Dae Choi, Pil Ja Seo, Uk Lee

**Affiliations:** a Department of Chemistry, Dongeui University, San 24 Kaya-dong, Busanjin-gu, Busan 614-714, Republic of Korea; b Department of Chemistry, Pukyong National University, 599-1 Daeyeon 3-dong, Nam-gu, Busan 608-737, Republic of Korea

## Abstract

In the title compound, C_21_H_22_O_3_S, the five-membered ring adopts an envelope conformation with the *ipso* atom deviating by 0.596 (2) Å from the plane through the rest of the ring atoms. The dihedral angle between the mean planes of the benzo­furan and *m*-tolyl moieties is 78.4 (1)°. In the crystal, mol­ecules related by a glide plane are linked *via* C—H⋯O hydrogen bonds into chains along the *a-*axis direction. These chains are in turn connected by C—H⋯π inter­actions into layers parallel to the *ac* plane.

## Related literature   

For background information and the crystal structures of related compounds, see: Choi *et al.* (2012 ▶); Seo *et al.* (2011 ▶).
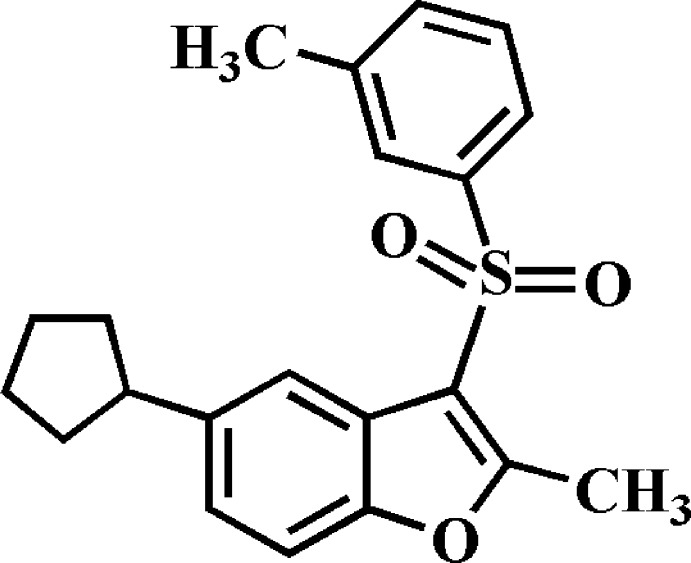


## Experimental   

### 

#### Crystal data  

C_21_H_22_O_3_S
*M* *_r_* = 354.45
Orthorhombic, 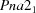

*a* = 18.2293 (7) Å
*b* = 6.1955 (3) Å
*c* = 15.9471 (8) Å
*V* = 1801.06 (14) Å^3^
*Z* = 4
Mo *K*α radiation
μ = 0.20 mm^−1^
*T* = 173 K
0.58 × 0.20 × 0.14 mm

#### Data collection  

Bruker SMART APEXII CCD diffractometer
Absorption correction: multi-scan (*SADABS*; Bruker, 2009 ▶) *T*_min_ = 0.683, *T*_max_ = 0.746
9604 measured reflections
3543 independent reflections
3115 reflections with *I* > 2σ(*I*)
*R*_int_ = 0.031

#### Refinement  

*R*[*F* ^2^ > 2σ(*F* ^2^)] = 0.045
*wR*(*F* ^2^) = 0.113
*S* = 1.04
3543 reflections
228 parameters
1 restraint
H-atom parameters constrained
Δρ_max_ = 0.54 e Å^−3^
Δρ_min_ = −0.24 e Å^−3^
Absolute structure: Flack (1983 ▶), 1205 Friedel pairs
Absolute structure parameter: 0.00 (9)

### 

Data collection: *APEX2* (Bruker, 2009 ▶); cell refinement: *SAINT* (Bruker, 2009 ▶); data reduction: *SAINT*; program(s) used to solve structure: *SHELXS97* (Sheldrick, 2008 ▶); program(s) used to refine structure: *SHELXL97* (Sheldrick, 2008 ▶); molecular graphics: *ORTEP-3 for Windows* (Farrugia, 2012 ▶) and *DIAMOND* (Brandenburg, 1998 ▶); software used to prepare material for publication: *SHELXL97*.

## Supplementary Material

Crystal structure: contains datablock(s) I. DOI: 10.1107/S1600536814006187/ld2123sup1.cif

Structure factors: contains datablock(s) I. DOI: 10.1107/S1600536814006187/ld2123Isup2.hkl

Supporting information file. DOI: 10.1107/S1600536814006187/ld2123Isup3.cml

CCDC reference: 992719

Additional supporting information:  crystallographic information; 3D view; checkCIF report

## Figures and Tables

**Table 1:** Hydrogen-bond geometry (Å, °) *Cg*1 is the centroid of the C2–C7 benzene ring.

*D*—H⋯*A*	*D*—H	H⋯*A*	*D*⋯*A*	*D*—H⋯*A*
C6—H6⋯O3^i^	0.95	2.49	3.263 (3)	139
C13—H13*B*⋯*Cg*1^ii^	0.99	2.99	3.671 (3)	127

Symmetry codes: (i) 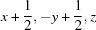; (ii) 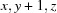.

**Fig. 1.**
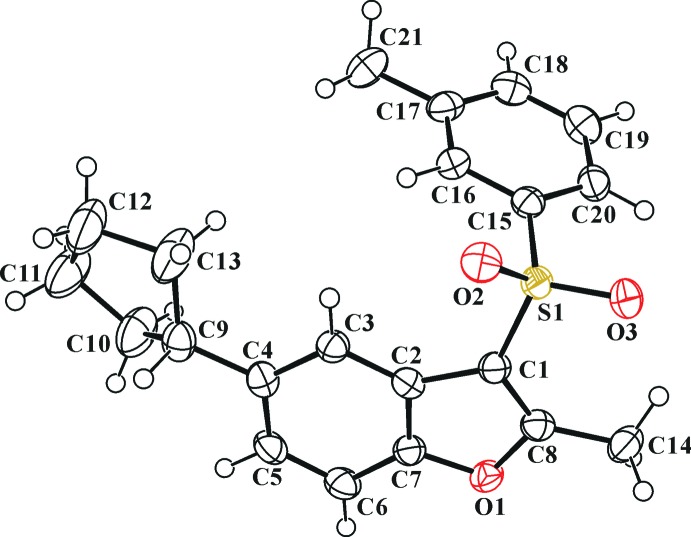
The molecular structure of the title molecule with the atom numbering scheme The displacement ellipsoids are drawn at the 50% probability level. The hydrogen atoms are presented as small spheres of arbitrary radius.

**Fig. 2.**
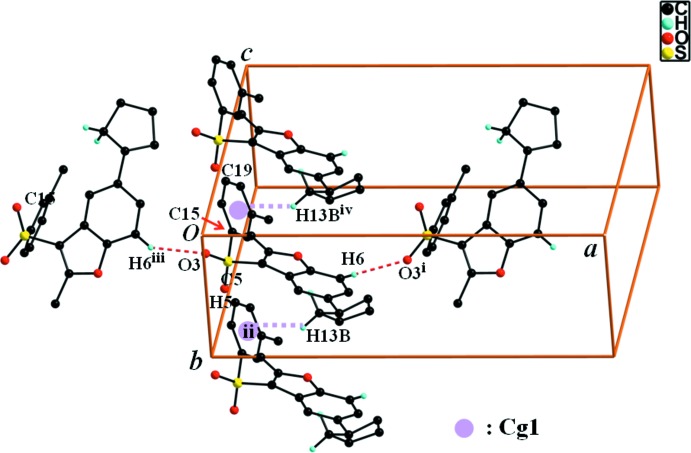
A view of the C—H···O and C—H···π interactions (dotted lines) in the crystal structure of the title compound. H atoms non-participating in hydrogen-bonding were omitted for clarity. [Symmetry codes: (i) x + 1/2, - y + 1/2, z; (ii) x, y + 1, z; (iii) x - 1/2, - y + 1/2, z; (iv) x, y - 1, z.]

## supplementary crystallographic information

### 1. Comment

As a part of our ongoing study of 5-cyclopentyl-2-methyl-1-benzofuran derivatives containing phenylsulfonyl (Seo *et al.*, 2011) and 4-bromophenylsulfonyl (Choi *et al.*, 2012) substituents in the 3-position, we report here on the crystal structure of the title compound.

In the title molecule (Fig. 1), the benzofuran ring system is essentially planar, with a mean deviation of 0.015 (2) Å from the least-squares plane defined by the nine constituent atoms. The 3-methylphenyl ring is essentially planar, with a mean deviation of 0.008 (2) Å from the least-squares plane defined by the six constituent atoms. The cyclopentyl ring has an envelope conformation. The dihedral angle formed by the benzofuran ring system and the 3-methylphenyl ring is 78.44 (8)°.

In the crystal structure (Fig. 2), the molecules are linked by C—H···O hydrogen bonds (Table 1) related by gliding plane a perpendicular to *b*-axis. The chains of C—H···O bonded molecules are stacked by C—H···π interactions (Table 1, Cg1 is the centroid of the C15–C20 3-methylphenyl-ring), resulting in a two-dimensional supramolecular layers.

### 2. Experimental

3-Chloroperoxybenzoic acid (77%, 448 mg, 2.0 mmol) was added in small portions to a stirred solution of 5-cyclopentyl-2-methyl-3-(3-methylphenylsulfanyl)-1-benzofuran (290 mg, 0.9 mmol) in dichloromethane (30 mL) at 273 K. After being stirred at room temperature for 10h, the mixture was washed with saturated sodium bicarbonate solution and the organic layer was separated, dried over magnesium sulfate, filtered and concentrated at reduced pressure. The residue was purified by column chromatography (benzene) to afford the title compound as a colorless solid [yield 73%, m.p. 417–418 K; *R*_f_ = 0.48 (benzene)]. Single crystals suitable for X-ray diffraction were prepared by slow evaporation of a solution of the title compound in acetone at room temperature.

### 3. Refinement

All H atoms were positioned geometrically and refined using a riding model, with C—H = 0.95 Å for aryl, 1.00 Å for methine, 0.99 Å for methylene and 0.98 Å for methyl H atoms, respectively. *U*_iso_ (H) = 1.2*U*_eq_ (C) for aryl, methine and methylene, and 1.5*U*_eq_ (C) for methyl H atoms. The positions of methyl hydrogens were optimized using the SHELXL-97's command AFIX 137 (Sheldrick, 2008).

### Crystal data

**Table:**

C_21_H_22_O_3_S	*D*_x_ = 1.307 Mg m^−^^3^
*M**_r_* = 354.45	Melting point = 418–417 K
Orthorhombic, *P**n**a*2_1_	Mo *K*α radiation, λ = 0.71073 Å
Hall symbol: P 2c -2n	Cell parameters from 3275 reflections
*a* = 18.2293 (7) Å	θ = 2.6–28.0°
*b* = 6.1955 (3) Å	µ = 0.20 mm^−^^1^
*c* = 15.9471 (8) Å	*T* = 173 K
*V* = 1801.06 (14) Å^3^	Block, colourless
*Z* = 4	0.58 × 0.20 × 0.14 mm
*F*(000) = 752	

### Data collection

**Table:**

Bruker SMART APEXII CCD diffractometer	3543 independent reflections
Radiation source: rotating anode	3115 reflections with *I* > 2σ(*I*)
Graphite multilayer monochromator	*R*_int_ = 0.031
Detector resolution: 10.0 pixels mm^-1^	θ_max_ = 28.4°, θ_min_ = 2.2°
φ and ω scans	*h* = −24→20
Absorption correction: multi-scan (*SADABS*; Bruker, 2009)	*k* = −8→8
*T*_min_ = 0.683, *T*_max_ = 0.746	*l* = −15→21
9604 measured reflections	

### Refinement

**Table:**

Refinement on *F*^2^	Secondary atom site location: difference Fourier map
Least-squares matrix: full	Hydrogen site location: difference Fourier map
*R*[*F*^2^ > 2σ(*F*^2^)] = 0.045	H-atom parameters constrained
*wR*(*F*^2^) = 0.113	*w* = 1/[σ^2^(*F*_o_^2^) + (0.0575*P*)^2^ + 0.6298*P*] where *P* = (*F*_o_^2^ + 2*F*_c_^2^)/3
*S* = 1.04	(Δ/σ)_max_ < 0.001
3543 reflections	Δρ_max_ = 0.54 e Å^−^^3^
228 parameters	Δρ_min_ = −0.24 e Å^−^^3^
1 restraint	Absolute structure: Flack (1983), 1205 Friedel pairs
Primary atom site location: structure-invariant direct methods	Absolute structure parameter: 0.00 (9)

### Special details

**Table:**

Geometry. All esds (except the esd in the dihedral angle between two l.s. planes) are estimated using the full covariance matrix. The cell esds are taken into account individually in the estimation of esds in distances, angles and torsion angles; correlations between esds in cell parameters are only used when they are defined by crystal symmetry. An approximate (isotropic) treatment of cell esds is used for estimating esds involving l.s. planes.
Refinement. Refinement of F^2^ against ALL reflections. The weighted R-factor wR and goodness of fit S are based on F^2^, conventional R-factors R are based on F, with F set to zero for negative F^2^. The threshold expression of F^2^ > 2sigma(F^2^) is used only for calculating R-factors(gt) etc. and is not relevant to the choice of reflections for refinement. R-factors based on F^2^ are statistically about twice as large as those based on F, and R- factors based on ALL data will be even larger.

### Fractional atomic coordinates and isotropic or equivalent isotropic displacement parameters (Å^2^)

**Table:**

	*x*	*y*	*z*	*U*_iso_*/*U*_eq_	
S1	0.04477 (3)	0.35807 (10)	0.10257 (5)	0.02864 (15)	
O1	0.23298 (10)	0.1800 (3)	0.00693 (12)	0.0330 (4)	
O2	0.03032 (9)	0.5860 (3)	0.10602 (17)	0.0364 (4)	
O3	−0.00242 (10)	0.2226 (3)	0.05328 (14)	0.0384 (5)	
C1	0.13426 (13)	0.3255 (4)	0.06741 (17)	0.0279 (5)	
C2	0.19660 (12)	0.4600 (4)	0.08929 (17)	0.0268 (6)	
C3	0.20851 (14)	0.6445 (4)	0.13714 (18)	0.0296 (6)	
H3	0.1686	0.7160	0.1636	0.035*	
C4	0.27969 (14)	0.7230 (4)	0.14570 (18)	0.0307 (6)	
C5	0.33751 (12)	0.6156 (4)	0.1049 (2)	0.0350 (6)	
H5	0.3859	0.6701	0.1110	0.042*	
C6	0.32665 (14)	0.4335 (5)	0.0562 (2)	0.0361 (6)	
H6	0.3659	0.3625	0.0285	0.043*	
C7	0.25515 (14)	0.3616 (4)	0.05046 (19)	0.0290 (5)	
C8	0.15930 (14)	0.1608 (4)	0.01931 (18)	0.0304 (6)	
C9	0.29660 (16)	0.9193 (5)	0.1986 (2)	0.0375 (6)	
H9	0.3094	1.0402	0.1597	0.045*	
C10	0.3623 (2)	0.8851 (7)	0.2584 (3)	0.0622 (11)	
H10A	0.3591	0.7436	0.2870	0.075*	
H10B	0.4094	0.8935	0.2277	0.075*	
C11	0.3549 (2)	1.0726 (8)	0.3217 (3)	0.0695 (12)	
H11A	0.3658	1.0215	0.3792	0.083*	
H11B	0.3896	1.1900	0.3076	0.083*	
C12	0.2784 (2)	1.1510 (7)	0.3164 (3)	0.0742 (14)	
H12A	0.2772	1.3006	0.2945	0.089*	
H12B	0.2550	1.1488	0.3724	0.089*	
C13	0.23894 (19)	0.9984 (8)	0.2568 (3)	0.0744 (15)	
H13A	0.2166	0.8769	0.2879	0.089*	
H13B	0.1999	1.0754	0.2256	0.089*	
C14	0.12535 (16)	−0.0293 (5)	−0.0228 (2)	0.0396 (7)	
H14A	0.0774	−0.0591	0.0026	0.059*	
H14B	0.1573	−0.1553	−0.0160	0.059*	
H14C	0.1190	0.0017	−0.0826	0.059*	
C15	0.04614 (13)	0.2588 (5)	0.20576 (18)	0.0293 (6)	
C16	0.07316 (15)	0.3898 (5)	0.26988 (19)	0.0325 (6)	
H16	0.0892	0.5319	0.2571	0.039*	
C17	0.07705 (15)	0.3171 (5)	0.35138 (19)	0.0356 (6)	
C18	0.05211 (15)	0.1092 (5)	0.3682 (2)	0.0392 (7)	
H18	0.0534	0.0564	0.4241	0.047*	
C19	0.02542 (16)	−0.0220 (5)	0.3047 (2)	0.0403 (7)	
H19	0.0085	−0.1631	0.3178	0.048*	
C20	0.02297 (14)	0.0489 (5)	0.2224 (2)	0.0351 (6)	
H20	0.0060	−0.0426	0.1787	0.042*	
C21	0.1076 (2)	0.4573 (6)	0.4203 (2)	0.0501 (8)	
H21A	0.0697	0.4824	0.4627	0.075*	
H21B	0.1234	0.5958	0.3967	0.075*	
H21C	0.1497	0.3849	0.4462	0.075*	

### Atomic displacement parameters (Å^2^)

**Table:**

	*U*^11^	*U*^22^	*U*^33^	*U*^12^	*U*^13^	*U*^23^
S1	0.0223 (2)	0.0339 (3)	0.0297 (3)	−0.0001 (2)	−0.0018 (3)	−0.0038 (3)
O1	0.0291 (9)	0.0375 (10)	0.0324 (11)	0.0016 (8)	0.0024 (8)	−0.0091 (9)
O2	0.0315 (8)	0.0360 (9)	0.0419 (11)	0.0067 (7)	−0.0011 (10)	−0.0003 (12)
O3	0.0270 (9)	0.0482 (11)	0.0399 (12)	−0.0067 (8)	−0.0046 (8)	−0.0062 (10)
C1	0.0255 (11)	0.0322 (13)	0.0259 (13)	0.0005 (10)	−0.0006 (10)	−0.0011 (11)
C2	0.0236 (10)	0.0308 (13)	0.0258 (15)	0.0002 (9)	−0.0010 (9)	0.0047 (11)
C3	0.0276 (12)	0.0312 (14)	0.0299 (15)	0.0006 (10)	0.0005 (10)	−0.0015 (12)
C4	0.0306 (12)	0.0320 (14)	0.0297 (15)	−0.0032 (11)	−0.0063 (11)	0.0053 (12)
C5	0.0251 (10)	0.0423 (14)	0.0374 (15)	−0.0066 (10)	0.0020 (14)	0.0026 (15)
C6	0.0267 (12)	0.0438 (15)	0.0379 (17)	0.0004 (11)	0.0062 (11)	−0.0012 (14)
C7	0.0285 (12)	0.0322 (13)	0.0263 (13)	0.0001 (10)	0.0008 (10)	−0.0004 (12)
C8	0.0300 (12)	0.0339 (14)	0.0275 (15)	0.0005 (11)	0.0010 (11)	0.0014 (11)
C9	0.0447 (15)	0.0306 (14)	0.0374 (17)	−0.0042 (12)	−0.0063 (13)	0.0038 (13)
C10	0.0471 (18)	0.071 (3)	0.068 (3)	0.0007 (18)	−0.0165 (18)	−0.026 (2)
C11	0.054 (2)	0.083 (3)	0.071 (3)	0.004 (2)	−0.016 (2)	−0.040 (2)
C12	0.054 (2)	0.071 (3)	0.098 (4)	−0.0056 (19)	−0.006 (2)	−0.048 (3)
C13	0.0405 (18)	0.081 (3)	0.102 (4)	0.0042 (18)	−0.008 (2)	−0.057 (3)
C14	0.0441 (16)	0.0411 (16)	0.0335 (17)	0.0001 (13)	−0.0023 (13)	−0.0110 (14)
C15	0.0230 (11)	0.0331 (13)	0.0317 (15)	0.0029 (10)	0.0012 (10)	−0.0022 (12)
C16	0.0326 (13)	0.0328 (15)	0.0320 (16)	−0.0003 (11)	0.0037 (12)	−0.0052 (12)
C17	0.0334 (13)	0.0420 (16)	0.0315 (16)	0.0059 (12)	0.0039 (12)	−0.0049 (13)
C18	0.0353 (14)	0.0450 (18)	0.0373 (18)	0.0044 (13)	0.0022 (12)	0.0063 (14)
C19	0.0374 (14)	0.0354 (16)	0.048 (2)	0.0006 (12)	0.0003 (14)	0.0080 (14)
C20	0.0292 (12)	0.0336 (14)	0.0426 (18)	−0.0025 (11)	0.0005 (12)	−0.0045 (13)
C21	0.062 (2)	0.056 (2)	0.0327 (18)	0.0023 (17)	−0.0065 (15)	−0.0102 (16)

### Geometric parameters (Å, º)

**Table:**

S1—O3	1.436 (2)	C11—C12	1.479 (5)
S1—O2	1.4374 (18)	C11—H11A	0.9900
S1—C1	1.737 (2)	C11—H11B	0.9900
S1—C15	1.757 (3)	C12—C13	1.522 (5)
O1—C8	1.363 (3)	C12—H12A	0.9900
O1—C7	1.382 (3)	C12—H12B	0.9900
C1—C8	1.356 (4)	C13—H13A	0.9900
C1—C2	1.451 (3)	C13—H13B	0.9900
C2—C7	1.376 (4)	C14—H14A	0.9800
C2—C3	1.392 (4)	C14—H14B	0.9800
C3—C4	1.392 (4)	C14—H14C	0.9800
C3—H3	0.9500	C15—C20	1.393 (4)
C4—C5	1.406 (4)	C15—C16	1.395 (4)
C4—C9	1.511 (4)	C16—C17	1.377 (4)
C5—C6	1.383 (4)	C16—H16	0.9500
C5—H5	0.9500	C17—C18	1.392 (4)
C6—C7	1.381 (4)	C17—C21	1.507 (4)
C6—H6	0.9500	C18—C19	1.388 (5)
C8—C14	1.490 (4)	C18—H18	0.9500
C9—C13	1.485 (5)	C19—C20	1.384 (5)
C9—C10	1.546 (5)	C19—H19	0.9500
C9—H9	1.0000	C20—H20	0.9500
C10—C11	1.545 (5)	C21—H21A	0.9800
C10—H10A	0.9900	C21—H21B	0.9800
C10—H10B	0.9900	C21—H21C	0.9800
			
O3—S1—O2	119.03 (12)	C12—C11—H11B	110.3
O3—S1—C1	108.55 (12)	C10—C11—H11B	110.3
O2—S1—C1	107.38 (11)	H11A—C11—H11B	108.6
O3—S1—C15	108.46 (13)	C11—C12—C13	106.1 (3)
O2—S1—C15	108.09 (14)	C11—C12—H12A	110.5
C1—S1—C15	104.38 (12)	C13—C12—H12A	110.5
C8—O1—C7	106.7 (2)	C11—C12—H12B	110.5
C8—C1—C2	107.7 (2)	C13—C12—H12B	110.5
C8—C1—S1	125.89 (19)	H12A—C12—H12B	108.7
C2—C1—S1	126.24 (19)	C9—C13—C12	105.1 (3)
C7—C2—C3	119.3 (2)	C9—C13—H13A	110.7
C7—C2—C1	104.2 (2)	C12—C13—H13A	110.7
C3—C2—C1	136.5 (2)	C9—C13—H13B	110.7
C2—C3—C4	119.1 (2)	C12—C13—H13B	110.7
C2—C3—H3	120.5	H13A—C13—H13B	108.8
C4—C3—H3	120.5	C8—C14—H14A	109.5
C3—C4—C5	119.2 (3)	C8—C14—H14B	109.5
C3—C4—C9	121.7 (3)	H14A—C14—H14B	109.5
C5—C4—C9	119.1 (2)	C8—C14—H14C	109.5
C6—C5—C4	122.6 (2)	H14A—C14—H14C	109.5
C6—C5—H5	118.7	H14B—C14—H14C	109.5
C4—C5—H5	118.7	C20—C15—C16	120.7 (3)
C7—C6—C5	115.8 (2)	C20—C15—S1	120.1 (2)
C7—C6—H6	122.1	C16—C15—S1	119.2 (2)
C5—C6—H6	122.1	C17—C16—C15	121.3 (3)
C2—C7—C6	124.0 (3)	C17—C16—H16	119.4
C2—C7—O1	111.1 (2)	C15—C16—H16	119.4
C6—C7—O1	124.9 (2)	C16—C17—C18	117.9 (3)
C1—C8—O1	110.4 (2)	C16—C17—C21	121.2 (3)
C1—C8—C14	135.2 (2)	C18—C17—C21	120.9 (3)
O1—C8—C14	114.4 (2)	C19—C18—C17	121.0 (3)
C13—C9—C4	118.0 (3)	C19—C18—H18	119.5
C13—C9—C10	102.0 (3)	C17—C18—H18	119.5
C4—C9—C10	113.1 (3)	C20—C19—C18	121.2 (3)
C13—C9—H9	107.7	C20—C19—H19	119.4
C4—C9—H9	107.7	C18—C19—H19	119.4
C10—C9—H9	107.7	C19—C20—C15	117.9 (3)
C11—C10—C9	103.5 (3)	C19—C20—H20	121.1
C11—C10—H10A	111.1	C15—C20—H20	121.1
C9—C10—H10A	111.1	C17—C21—H21A	109.5
C11—C10—H10B	111.1	C17—C21—H21B	109.5
C9—C10—H10B	111.1	H21A—C21—H21B	109.5
H10A—C10—H10B	109.0	C17—C21—H21C	109.5
C12—C11—C10	106.9 (3)	H21A—C21—H21C	109.5
C12—C11—H11A	110.3	H21B—C21—H21C	109.5
C10—C11—H11A	110.3		
			
O3—S1—C1—C8	−17.0 (3)	C7—O1—C8—C1	1.2 (3)
O2—S1—C1—C8	−146.9 (2)	C7—O1—C8—C14	−179.5 (2)
C15—S1—C1—C8	98.5 (3)	C3—C4—C9—C13	13.6 (5)
O3—S1—C1—C2	168.0 (2)	C5—C4—C9—C13	−165.6 (3)
O2—S1—C1—C2	38.1 (3)	C3—C4—C9—C10	132.5 (3)
C15—S1—C1—C2	−76.5 (3)	C5—C4—C9—C10	−46.7 (4)
C8—C1—C2—C7	0.5 (3)	C13—C9—C10—C11	−36.3 (4)
S1—C1—C2—C7	176.3 (2)	C4—C9—C10—C11	−164.1 (3)
C8—C1—C2—C3	−178.2 (3)	C9—C10—C11—C12	19.1 (5)
S1—C1—C2—C3	−2.4 (5)	C10—C11—C12—C13	5.2 (6)
C7—C2—C3—C4	−1.1 (4)	C4—C9—C13—C12	164.8 (3)
C1—C2—C3—C4	177.5 (3)	C10—C9—C13—C12	40.2 (4)
C2—C3—C4—C5	0.8 (4)	C11—C12—C13—C9	−28.9 (5)
C2—C3—C4—C9	−178.4 (3)	O3—S1—C15—C20	17.8 (2)
C3—C4—C5—C6	0.0 (5)	O2—S1—C15—C20	148.1 (2)
C9—C4—C5—C6	179.2 (3)	C1—S1—C15—C20	−97.8 (2)
C4—C5—C6—C7	−0.5 (5)	O3—S1—C15—C16	−164.5 (2)
C3—C2—C7—C6	0.5 (4)	O2—S1—C15—C16	−34.2 (2)
C1—C2—C7—C6	−178.4 (3)	C1—S1—C15—C16	79.9 (2)
C3—C2—C7—O1	179.2 (2)	C20—C15—C16—C17	−0.7 (4)
C1—C2—C7—O1	0.2 (3)	S1—C15—C16—C17	−178.3 (2)
C5—C6—C7—C2	0.3 (5)	C15—C16—C17—C18	−0.9 (4)
C5—C6—C7—O1	−178.2 (3)	C15—C16—C17—C21	178.8 (3)
C8—O1—C7—C2	−0.9 (3)	C16—C17—C18—C19	1.2 (4)
C8—O1—C7—C6	177.7 (3)	C21—C17—C18—C19	−178.6 (3)
C2—C1—C8—O1	−1.1 (3)	C17—C18—C19—C20	0.3 (5)
S1—C1—C8—O1	−176.91 (19)	C18—C19—C20—C15	−1.9 (4)
C2—C1—C8—C14	179.9 (3)	C16—C15—C20—C19	2.1 (4)
S1—C1—C8—C14	4.1 (5)	S1—C15—C20—C19	179.7 (2)

### Hydrogen-bond geometry (Å, º)

Cg1 is the centroid of the C2–C7 benzene ring.

**Table:**

*D*—H···*A*	*D*—H	H···*A*	*D*···*A*	*D*—H···*A*
C6—H6···O3^i^	0.95	2.49	3.263 (3)	139
C13—H13*B*···*Cg*1^ii^	0.99	2.99	3.671 (3)	127

Symmetry codes: (i) *x*+1/2, −*y*+1/2, *z*; (ii) *x*, *y*+1, *z*.
